# Aflatoxin B_1_-Adsorbing Capability of *Pleurotus eryngii* Mycelium: Efficiency and Modeling of the Process

**DOI:** 10.3389/fmicb.2019.01386

**Published:** 2019-06-25

**Authors:** Miriam Haidukowski, Eliana Casamassima, Maria Teresa Cimmarusti, Maria Teresa Branà, Francesco Longobardi, Pasquale Acquafredda, Antonio Logrieco, Claudio Altomare

**Affiliations:** ^1^Department of Biology, Agriculture and Food Science, Institute of Sciences of Food Production, National Research Council (CNR), Bari, Italy; ^2^Department of Chemistry, University of Bari, Bari, Italy; ^3^Department of Earth and Geo-Environmental Sciences, University of Bari, Bari, Italy

**Keywords:** biosorption, aflatoxin, *Pleurotus eryngii*, feed additive, king oyster mushroom

## Abstract

Aflatoxin B_1_ (AfB_1_) is a carcinogenic mycotoxin that contaminates food and feed worldwide. We determined the AfB_1_-adsorption capability of non-viable *Pleurotus eryngii* mycelium, an edible fungus, as a potential means for removal of AfB_1_ from contaminated solutions. Lyophilized mycelium was produced and made enzymatically inert by sterilization at high temperatures. The material thus obtained was characterized by scanning electron microscopy with regard to the morpho-structural properties of the mycotoxin-adsorbing surfaces. The active surfaces appeared rough and sponge-like. The AfB_1_-mycelium system reached equilibrium at 37°C, 30 min, and pH 5–7, conditions that are compatible with the gastro-intestinal system of animals. The system remained stable for 48 h at room temperature, at pH 3, pH 7, and pH 7.4. A thermodynamic study of the process showed that this is a spontaneous and physical adsorption process, with a maximum of 85 ± 13% of removal efficiency of AfB_1_ by *P. eryngii* mycelium. These results suggest that biosorbent materials obtained from the mycelium of the mushroom *P. eryngii* could be used as a low-cost and effective feed additive for AfB_1_ detoxification.

## Introduction

The contamination of food with mycotoxins is a worldwide problem with impact on the health of humans and animals and on the economy of many countries, especially in sub-tropical and temperate areas. The problem is caused by the spoilage of agricultural products by microscopic filamentous fungi, mostly belonging to species and strains in the genera *Aspergillus, Fusarium,* and *Penicillium*, which in favorable environmental conditions are able to produce toxic secondary metabolites that accumulate into food and feedstuffs. Aflatoxin B_1_ (AfB_1_) is the most toxic mycotoxin and is classified in the “group 1” substances (carcinogenic to humans) by the International Agency for Research on Cancer (World Health Organization and International Agency for Research on Cancer, 1993). AFB_1_ has potent hepatotoxic, carcinogenic, and mutagenic effects on humans and animals and is produced mainly by isolates of the species *Aspergillus flavus* and *A. parasiticus*. AFB_1_ occurrence is a major problem in a number of crops, including cereals, groundnuts, legumes, and cotton seeds, which can be contaminated at any stage from field to storage. Human exposure to AFB_1_ can result from ingestion of contaminated food or from consumption of meat, eggs, and dairy products from animals that have been fed with contaminated feed (Rushing and Selim, 2019).

Much attention is devoted to measures of prevention and monitoring that aim at the reduction of contamination levels in commodities. Nevertheless, often the contamination occurs despite careful application of prevention means, and it is necessary to put into action decontamination measures to avoid the complete loss of the produce and mitigate the risk of mycotoxins in food and feed. Different chemical and physical methods for decontamination and detoxification have been developed, but their use is often limited by high cost, lack of information on nature and toxicity of degradation products and, above all, loss of nutritional, organoleptic, and visual qualities (Boudergue et al., 2009).

For protection of animals from aflatoxicosis, the use of adsorbent materials which are able to bind with high efficiency the mycotoxins in feeds is being receiving growing interest (Williams et al., 2004). The adsorbents reduce the bioavailability of mycotoxins in the gastro-intestinal tract and thus their diffusion into the bloodstream and transport to the target organs (Kabak et al., 2006; Kolosova and Stroka, 2011). Aluminosilicates are the most used adsorbents, followed by activated carbon and special polymers (Huwig et al., 2001; Vila-Donat et al., 2018). The EU has approved the use of various adsorbent materials as food additives, for example the use of bentonite as a feed additive for all animal species is regulated by the Commission Implementing Regulation (EU) No. 1060/2013. The efficiency of these mycotoxin ligands differs considerably, depending on the chemical structures of both the adsorbent and the toxin (Fu and Viraraghavan, 2001; Crini, 2006). Also, the use of these materials may have a negative impact on the quality of decontaminated feeds. For this reason, scientific interest is partly shifting toward the use of less expensive, though effective, and environmentally friendly materials such as microbial biomasses (Low et al., 2008).

Some studies have demonstrated the ability of some strains of lactic and bifidus bacteria to efficiently bind AfB_1_ (El-Nezami et al., 1998; Peltonen et al., 2001), through a chemical-physical phenomenon related to the features of structural elements of the bacterial wall such as peptiglycans and polysaccharides (Kabak et al., 2006). However, few materials have been studied for this purpose and to our knowledge there are no studies concerned with the use of non-viable fungal mycelium as mycotoxin adsorbent. The mycelium of fungi has noteworthy adsorbing properties, mostly due to the ability of the polysaccharides constituting the cell wall to form hydrogen, ionic, or hydrophobic interactions with organic and inorganic molecules (Huwig et al., 2001). These properties are the subject of research with practical applications in different contexts, including bioremediation of soils and wastewater from heavy metals and organic pollutants (Gavrilescu, 2004; Gadd, 2009). Hence, the use of fungal mycelium also as biosorbent for mycotoxins appears conceivable.

The present work is focused on a new adsorbent material made from mushroom mycelium. This novel biosorbent is different from those already on the market, since it is palatable and has nutritional value. Particularly, we report on the characterization of the biosorbent properties of the mycelium of *Pleurotus eriyngii* (DC.) Quél. (king oyster mushroom). This fungus combines several advantageous features. Species of *Pleurotus* can be grown easily and are cultivated worldwide. They can be grown on a variety of lignocellulosic materials, including wastes which are produced through agricultural, forest, and food-processing activities. They grow faster than other cultivable mushrooms and cultivation has no particular technical hurdles (Sánchez, 2010); therefore, large biomasses of *Pleurotus* spp. can be obtained at sensible cost. Besides being easily cultivable and edible, *Pleurotus* spp. exhibit some properties of biotechnological interest. These fungi are high producers of extracellular ligninolytic enzymes, namely phenol oxidases (mainly laccases) and peroxidases (lignin peroxidase and Mn peroxidase) (Sánchez, 2010). Because of the low substrate specificity of these ligninolytic enzymes, applications of *Pleurotus* have been investigated for bioremediation purposes, e.g., decontamination of wastewater and water sediments from phenolic endocrine disruptors (Loffredo et al., 2013), degradation of dyes (Kalmiş et al., 2008) and mycotoxins (Alberts et al., 2009; Branà et al., 2017) and of other recalcitrant environmental pollutants (Rigas et al., 2009; Purnomo et al., 2010). In addition, mycelia of *Pleurotus* spp. have been reported to have binding and sequestering capabilities for heavy metals (recently reviewed by Kapahi and Sachdeva, 2017).

Fungal cell walls have already received attention as biosorbents for bioremediation of polluted soils and wastewaters (Wang and Chen, 2009). We here propose a novel application intended for feed industry. In particular, we have investigated the capability of non-viable mycelium of *P. eryngii* to bind AfB_1_; in addition, the effects of physical and chemical conditions on the binding efficiency were studied through a Design of Experiment (DOE) methodology. In most of the works concerned with the adsorption process, an approach that takes into account one factor at a time is used, while there are few studies that use a factorial design model to evaluate the relative importance and the interaction of different operative factors on the biosorption process (Manal, 2007). The design determines which factors have significant effects on the response, as well as the cases in which the effect of a factor varies with the level of another (Brasil et al., 2006), using the least possible number of experiments. The determination of interactions between factors is the key for optimization of complex processes. In the absence of such a study, important interactions might remain undetermined and the optimization becomes difficult to achieve (Brasil et al., 2006). For this reason, our study firstly evaluated the effects of different factors on the adsorption process and then we proceeded with the assessment of the stability of the mycelium-mycotoxin system and the identification of the experimental conditions that achieve the highest efficiency in mycotoxin binding of AfB1 and its removal from a solution. Our results show that in the optimized process, non-viable mycelium of the fungus *P. eryngii* is able to absorb up to 85% of AfB_1_ at temperature (37°C) and pH (5 and 7) conditions that are compatible with animal physiology, and a possible development of fungal mycelium-based biosorbent as feed additive can be conceived.

## Materials and Methods

### Reagents and Standards

The standard solution of AfB_1_ at 1 mg/ml was prepared by dissolving the solid commercial mycotoxin (SigmaAldrich, Milan, Italy) in toluene/acetonitrile (9,1, v/v). The stock solution was diluted, at a concentration of 10 μg/ml and quantified according to AOAC Official Method 971.22 (AOAC, 2000). The stock solution was evaporated at 50°C in an air stream and dissolved in appropriate buffers (pH 5 or 7) at a concentration of 500 ng/ml. The calibration solutions were obtained by diluting at 0.6, 1.2, 2.4, 5.7, 11.0, 23.0, 57.0 ng/ml. The solutions were stored at −20°C and warmed to room temperature before use. All solvents (grade HPLC) were purchased by VWR. International S.r.l (Milan, Italy), water was of Milli-Q quality (Millipore, Bedford, MA, USA). Regenerated cellulose membrane filters (RC 0.2 μm) were obtained from Phenomenex (Bologna, Italy). The filter paper used was Whatman # 4 (Whatman, Maidstone, UK). The 0.1 mol/L phosphate buffer (PBS) was prepared by dissolving the tablets (SigmaAldrich, Milan, Italy) in water and adjusted to pH 7 or to pH 7.4 with sodium hydroxide. The 0.01 mol/L acetate buffer (pH 5) was prepared by dissolving tri-hydrate sodium acetate (SigmaAldrich, Milan, Italy) in water adjusted to pH 5 with acetic acid. The 1 mmol/L citrate buffer (pH 3) was prepared by dissolving tri-sodium citrate 2-hydrate in water and adjusted to pH 3 with citric acid.

### Preparation of the *Pleurotus eryngii* Mycelium

The isolate *P. eryngii* ITEM 13681 that was used in this study was obtained from the collection of Institute of Sciences of Food Production (ITEM Collection, http://www.ispa.cnr.it/Collection/, Bari, Italy). The culture was grown in purity on malt extract agar (MEA, Oxoid, Basingstoke, UK) slants, for 30 days at 28°C, which were used as sources of inoculum for subsequent cultures in malt extract broth (MEB). Five mycelial plugs (8 mm diameter) were transferred onto Roux flasks filled with 200 ml of MEB and incubated under static conditions for 20 days at 28°C. After incubation, the mycelium was separated from the culture broth by filtration through filter paper by applying vacuum and then washed four times with 25 ml of sterile distilled water. The biomass collected was then inactivated by autoclaving at 121°C for 20 min, lyophilized for 3 days, varying the temperature from −20 to 20°C and maintaining the pressure of 0.030 mbar, and finally ground with a mortar and then sieved to collect a fine powder (particle size ≤500 μm).

### Dosage of Laccase Activity

A 100 mmol/L sodium malonate buffer (pH 4.5) was prepared by dissolving sodium malonate hydrate in distilled water. The solution was adjusted to pH 4.5 with 100 mmol/L malonic acid. An amount of 0.1 gram of ground autoclaved mycelium was extracted with 5 ml of 100 mmol/L phosphate buffer (PBS) at pH 7.3 and incubated for 60 min, at 25°C in a rotary shaker at 150 rpm. The extract obtained was filtered and used for the enzymatic assay. The laccase activity was determined spectrophotometrically by oxidation of 2,2′-azino-bis-3-ethylbenzothiazoline-6-sulfonate (ABTS) at 37°C (Li et al., 2008). The reaction mixture (1.5 ml) contained 0.75 ml of sodium malonate buffer (100 mM, pH 4.5), 0.075 ml of ABTS (2 mM in water solution), 0.655 ml of H_2_O, and 0.02 ml of enzyme extract. The oxidation of the ABTS was evaluated spectrophotometrically (Varian Cary 50) by the increase of absorbance at 420 nm. One laccase unit was defined as the quantity of enzyme able to oxidize 1 μmol of ABTS in 1 min, given a molar extinction coefficient *ε*_420_ = 36,000 M^−1^ cm^−1^.

### Analysis of Aflatoxin B_1_

The chromatographic analysis of the AfB_1_ was performed by high-performance liquid chromatography (HPLC, Agilent Technology Series 1,260) associated to a fluorescence detector (FLD). Before the injection, the mycotoxin was derivatized by a photochemical post-column derivatization reaction (UVE™ LCTech GmbH, Obertaufkirchen, Germany). A Synergi 4U MAX-RP 80A reverse phase column (150 mm × 4.6 mm, 4.0 μm) (Phenomenex, Torrance, California, USA) was used, preceded by a pre-column (MAX-RP, 4 mm × 3.0 mm, Phenomenex) thermostatically controlled at 40°C. The mobile phase consisted of water-acetonitrile, 60:40, with a flow rate set at 1 ml/min. The fluorometric detector was set at the wavelengths of 365 nm (excitation) and 435 nm (emission). Under these analytical conditions, the retention time of the AfB_1_ was about 6 min. AfB_1_ was quantified by measuring the peak area and comparing it with the calibration curve obtained with standard solutions. The quantification limit of the method (LOQ) was 0.6 ng/ml, based on a 10:1 signal-to-noise ratio.

### Determination of Major Variables Affecting Adsorption and Optimization of the Process

A factorial design was employed to reduce the total number of experiments needed to achieve the optimization of the system. The design adopted determined which factors have significant effects on the response and how the effect of one factor varies with the levels of the other factors (interactions). A full factorial design 2^4^ was adopted. All the experiments were done in duplicate, the experiments were arranged in random blocks to avoid systematic errors, and the experiments were performed in two different working days. The variables studied were pH of solution (5 and 7), time of interaction t (30 and 120 min), mass of adsorbent (50 and 500 mg), and concentration of AfB_1_ (50 and 500 ng/ml). In order to evaluate both the stability of AfB_1_ in the buffer solutions under the experimental conditions and any nonspecific interaction of the toxin with the buffer components or the test tube surface, we prepared blank controls. A blank control consisted of a standard working solution of AfB_1_ in the absence of adsorbent material, which was treated in the same way as the experimental treatments. In addition, negative controls (solution containing the adsorbent material in the absence of AfB_1_) were set up during each test to assess the absence of potential matrix constituents that could interfere with the chromatographic analysis. Reduction of AfB_1_ in the treatments was compared to the blank control. The values of *p* from the analysis of variance (ANOVA) were used to check the significance (*p* < 0.05) of the effect of different parameters and of the interactions between variables (Kavak, 2009). Optimization of the process was carried out, considering the two most significant parameters obtained from the previous analysis: mass of adsorbent (m) and concentration of AfB_1_. A completely randomized factorial experimental design 3^2^ was used for optimization, in which the mass of adsorbent (400, 700, 1,000 mg) and AfB1 concentration (50, 525, 1,000 ng/ml) were investigated at three levels and five center points. Blank controls and negative controls were set up for this experiment as described above.

### Aflatoxin Adsorption Experiments

Different amounts of powdered mycelium were transferred into 15-ml test tubes and 8 ml of AfB_1_ solution at different concentrations at pH 5 or 7 were added. The suspensions were mixed on a vortex to ensure homogeneity and placed in an orbital shaker at 250 rpm, in the dark, at different temperatures and for different periods of time. Subsequently, the samples were centrifuged for 10 min at 10397 × *g* at 25°C, the supernatant was recovered, and the pellet was washed twice with the same buffer used for suspension. The supernatant and the washing solutions were collected and analyzed by HPLC/FLD. For each experiment, a control was prepared using AfB_1_ standard solution in buffer without adsorbent material, in order to evaluate the stability of mycotoxins in the buffer solution under the experimental conditions or the occurrence of any nonspecific toxin interaction with the surface of the tubes. A negative control consisting of the buffer solution without the adsorbent material and AfB_1_ was also analyzed to evaluate the absence of potential matrix constituents able to interfere with the chromatographic analysis of the toxin. The experiments were carried out in triplicate.

The percentage of adsorption (Ads%) was calculated using the following equation:

$$Ads\%=\frac{\left(C_{0}-C_{e}\right)}{C_{0}}\times 100$$

where C_0_ was the initial concentration of AfB_1_ in solution and C_e_ was the mycotoxin concentration measured in the supernatant and the washing solutions after the adsorption.

### Desorption

Aliquots of *P. eryngii* powdered mycelium were weighed and subjected to the treatment to assess the adsorption of AfB_1_. After recovery of the supernatant and the washing solutions, the remaining pellet was treated with 8 ml of either citrate buffer (pH 3) or phosphate-buffered saline buffer (PBS, pH 7.4). The tubes were kept at room temperature in the dark for 48 h and subsequently centrifuged for 10 min at 9500 × *g* at 25°C. The supernatant was recovered and analyzed by HPLC/FLD. The experiments were carried out in triplicate.

The percentage of desorption was determined by comparing the quantity of mycotoxin released (q_des_) and that adsorbed (q_a_) on mycelium, according to the following equation:

$$\%D=\frac{q_{des}}{q_{a}}\cdot 100$$

The mycotoxin released (q_des_) per gram of biomass was calculated from the concentration of mycotoxin after desorption (C_des_):

$$q_{des}=C_{des}\frac{V}{m}$$

where V was the volume of the solution and m was the weight of the biosorbent.

To test the biosorbent for re-usability, the AfB_1_ adsorbed was then extracted with methanol. The tubes were kept at 40°C in the dark for 1 h and subsequently centrifuged for 10 min at 8422 × *g* at 25°C. The supernatant was recovered and analyzed by HPLC/FLD.

### Adsorption Isotherms

The adsorption isotherms were determined to study the effect of the amount of adsorbent (isotherm I) and of the AfB_1_ concentration (isotherm II) on the mycotoxin binding. Equilibrium experiments were set up according to the result of a preliminary screening, using 30 min of contact time at pH 7. For isotherm I, the concentration of AfB_1_ was 200 ng/ml and the amount of mycelium varied from 600 to 1,200 mg. For isotherm II, the amount of mycelium was 250 mg and the concentration of AfB_1_ varied from 200 to 2000 ng/ml.

The amount of adsorbed mycotoxin (q_a_) ng of mycotoxin absorbed per milligram of fungal mycelium (ng/mg) was calculated as the difference between the concentration of mycotoxin in the test solution (C_0_) and the concentration of mycotoxin recovered from the supernatant of (C_e_), according to the following equation:

$$q_{a}=\left(\frac{C_{0}-C_{e}}{m}\right)\cdot V$$

where V was the volume of solution (ml) and m was the mass of fungal mycelium (mg).

The adsorption isotherms were obtained by plotting the values of the amount of mycotoxin adsorbed in mg/g at equilibrium (q_a_) as a function of the amount of residual mycotoxin in solution in ng/ml at equilibrium (C_e_), and reporting the percentage of adsorption as a function of the dosage of the adsorbent in mg/ml. The data were fitted by the Langmuir and Freundlich isotherm models (Freundlich, 1906; Langmuir, 1916).

A dimensionless constant known as the separation factor (K_R_) derived from the Langmuir (K_L_ is the Langmuir constant) equation was used to assess the favorability of adsorption:

$$K_{R}=\frac{1}{1+K_{L}C_{0}}$$

The Gibbs free energy change (ΔG^0^, kJ/mol), the standard enthalpy (ΔH^0^, kJ/mol), and the standard entropy (ΔS^0^, kJ/mol·K) were calculated according to Kavak, 2009.

### SEM Characterization

For scanning electron microscope (SEM) investigations, the samples were previously fixed on an aluminum stub with a carbon-based, electrically conductive, double-sided adhesive disc and then sputtered with a 30-nm-thick carbon film using an Edwards Auto 306 thermal evaporator.

Images of the samples were taken with a secondary electrons (SE) detector mounted on a SEM of LEO, model EVO50XVP. Operating conditions of the SEM were: 7.5 kV accelerating potential, 500 pA probe current, and 9 mm working distance.

### Statistics

Adsorption/desorption experiments were performed in triplicate. The results obtained were subjected to one-way ANOVA with a significance level of *p <* 0.05. The data-processing software used were Excel 2016 (Microsoft Corporation, Redmond, Washington, USA) and OriginPro 2017 (OriginLab Corporation, Northampton, Massachusetts, USA). The statistical software used was STATGRAPHICS® centurion XVII (Statpoint Technologies, Inc. The Plains, Virginia, USA).

## Results

### Laccase Activity in the Autoclaved Mycelium

In order to rule out the occurrence of enzymatic degradation in the reduction of AfB1 concentration in the solutions exposed to the adsorbent, ground mycelium of *P. eryngii* was autoclaved to obtain denaturation of the proteins and subsequently extracted with PBS (pH 7.3); the extract was then analyzed for laccase activity. No laccase activity was found in the *P. eryngii* mycelium subjected to the heat treatment. This allowed to clarify that the removal of AfB_1_ in the working solutions treated with autoclaved *P. eryngii* mycelium was not due to enzymatic degradation.

### Identification of Major Variables Affecting Adsorption

**Figure 1:**
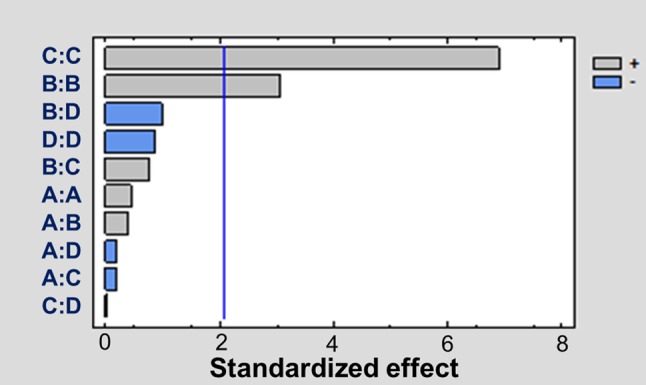
Pareto chart of the standardized effect for AfB_1_ adsorption. A is the pH, B is the adsorbent mass, C is the mycotoxin concentration, and D is the time. The effect of one factor is statistically significant (*p* < 0.05) if its absolute value is higher than 2.3 (sector of the chart at the right of the vertical line).

The ANOVA was employed to analyze the role of different variables (pH, time, mass of the adsorbent, and concentration of AfB1) on the adsorption process. The main factors and interaction effects are shown in Table 1. Only two factors, that is, mass of adsorbent and concentration of AfB_1_, were significantly different from 0 at the 95.0% confidence level (*p* < 0.05). Time, pH, and interaction between factors were not statistically significant. The Pareto chart of standardized effects at *p* = 0.05 is presented in Figure 1. The same two factors (mass of adsorbent and concentration of AfB1) showed a statistically significant effect (*p* = 0.05), with absolute values higher than 2.3.

**Table 1:** Effect of pH, mass of mycelium (m), AfB1 concentration (AfB1), and time (t), and interactions thereof on AfB_1_ adsorption by *P. eryngii* mycelium.

Source	Sum of squares	Df	Mean square	F-ratio	*p*
pH	69.0313	1	69.0313	0.20	0.6613
m	3260.28	1	3260.28	9.34	0.0062
AfB_1_	16607.5	1	16607.5	47.57	0.0000
t	247.531	1	247.531	0.71	0.4097
pH × m	57.7813	1	57.7813	0.17	0.6885
pH × AfB_1_	11.2813	1	11.2813	0.03	0.8591
pH × t	13.7813	1	13.7813	0.04	0.8445
m × AfB_1_	205.031	1	205.031	0.59	0.4524
m × t	344.531	1	344.531	0.99	0.3324
AfB_1_ × t	0.03125	1	0.03125	0.00	0.9925
blocks	38.2813	1	38.2813	0.11	0.7440
Total error	6982.13	20	349.106		
Total (corr.)	27837.2	31			

The effects are statistically significant if *p* < 0.05 (95% confidence level).

### Optimization of Adsorption

Mass of adsorbent (*m*) and concentration of AfB_1_ were identified as effective factors of adsorption and their effect was optimized by a factorial experiment in which the two variables were investigated at three levels. The 3^2^ factorial design matrix and the results of the experiments are shown in Table 2.

**Table 2:** The coded values for experimental design and the results.

Run	Mycelium (mg)	AfB1 (ng/ml)	Absorption (%)
1	1,000	525	62
2	700	525	58
3	700	1,000	59
4	700	525	59
5	1,000	50	96
6	700	525	61
7	700	525	61
8	400	1,000	46
9	700	50	72
10	400	50	78
11	700	525	62
12	400	525	53
13	1,000	1,000	60
14	700	525	60

The model expressed by Eq. (1), where the variables are expressed in their original units, represents the removal efficiency of AfB_1_ (*Ads %*) as a function of m and AfB_1_.

(1) $$\begin{array}{l} Ads\%=\;\;70.1995+0.0154816m-0.0640237AfB_{1}\\ \;\;\;\;\;\;\;\;\;\;\;\;\;\;\;\;\;\;+\;\;0.00000784314m^{2}-0.00000701754mAfB_{1}\\ \;\;\;\;\;\;\;\;\;\;\;\;\;\;\;\;\;\;+\;\;0.0000385856AfB_{1}{}^{2} \end{array}$$

The model equation is useful in indicating the direction in which the variables should be changed in order to optimize the AfB_1_-removal efficiency of the adsorbent. The results of ANOVA are presented in Table 3. The statistical significance of each coefficient was determined by values of *p*: the smaller the values of *p*, the more significant is the coefficient. This implies that the first-order main effects of mass of adsorbent and mycotoxin concentration are more significant than their quadratic main effect. However, the quadratic main effect of AfB_1_ concentration is more significant than other second main effect.

**Table 3:** Statistical significance of coefficients assessed by ANOVA.

Source	Sum of squares	Df	Mean square	F-ratio	*p*
m	280.167	1	280.167	10.65	0.0115
AfB_1_	1093.5	1	1093.5	41.57	0.0002
m^2^	1.41176	1	1.41176	0.05	0.8226
mAfB_1_	4.0	1	4.0	0.15	0.7067
AfB_1_^2^	214.745	1	214.745	8.16	0.0212
Total error	210.422	10	26.3027		
Total (corr.)	1867.21	13			

The fit of the model was checked by the determination of the coefficient (*R^2^*). In this case, the value of the determination coefficient (*R^2^* = 0.8873) indicated that the 11.27% of the total variable was not explained by the model.

**Figure 2:**
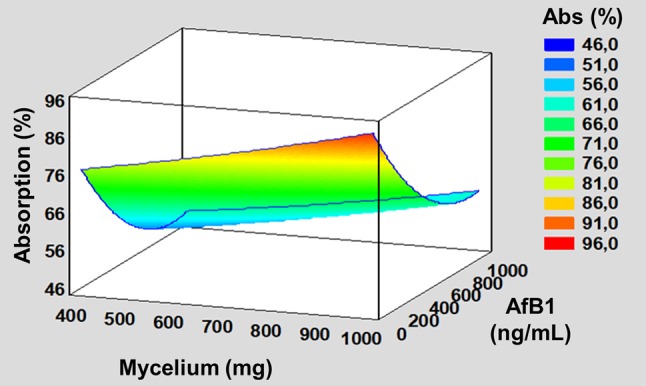
Estimate response surface plot for the effect of mass of adsorbent and mycotoxin concentration on the AfB_1_ removal.

Figure 2 shows the effect of the initial concentration of AfB_1_ and the quantity of the mycelium on mycotoxin removal efficiency.

The working conditions at the optimum point for removal efficiency of AfB_1_ were determined as follows:

m=1000mg

$${\mathrm{AfB}}_{1}=50\;\mathrm{ng}/\mathrm{ml}$$

Application of the optimum parameter *m* = 1,000 mg and AfB_1_ = 50 ng/ml to our model resulted in a theoretical optimum removal efficiency of AfB_1_ by *Pleurotus* mycelium of 90.07%. The experimentally determined removal efficiency for the same levels of “*m*” and “AfB_1_” was 85 ± 13% showing a satisfactory goodness-to-fit of the model.

### SEM Analysis

**Figure 3:**
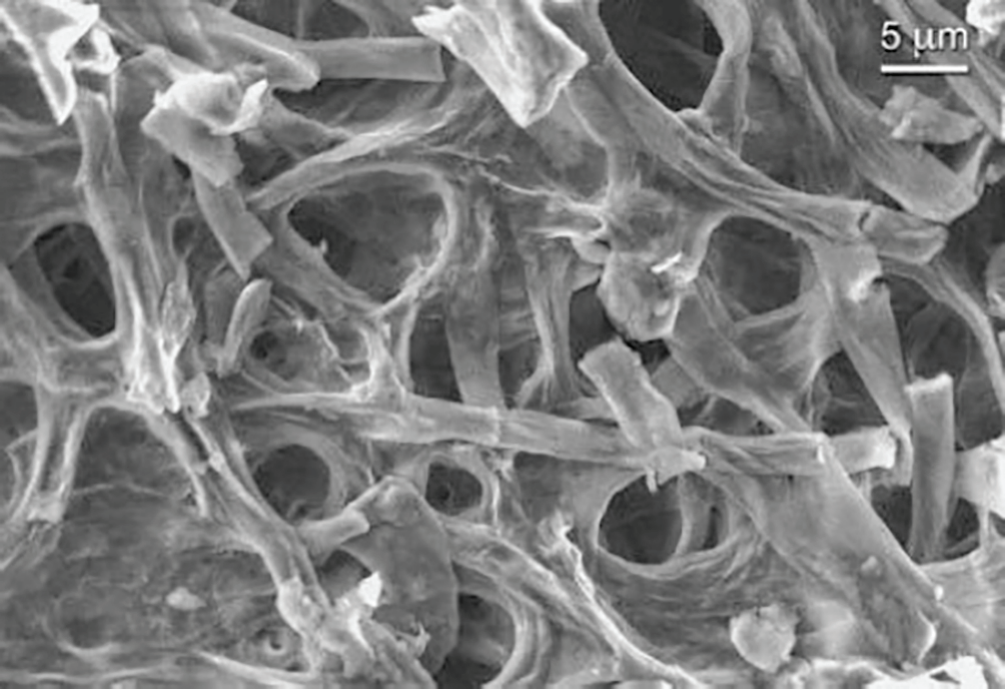
SEM secondary electron micrograph of *P. eryngii* mycelium.

A SEM micrograph of the *P. eryngii* mycelium is shown in Figure 3. The surface appears rough and sponge-like. The approximate pore size of 5–15 μm was measured from SEM analysis.

### Adsorption Isotherms

**Figure 4:**
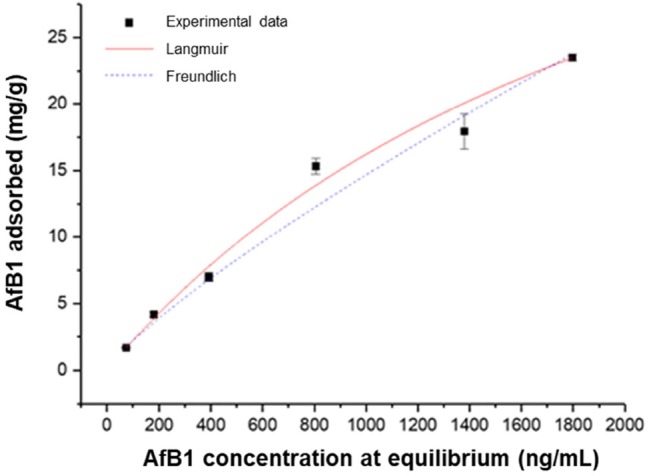
Effect of mycotoxin concentration on AfB_1_ adsorption by mycelium. Equilibrium adsorption isotherms were obtained at constant temperature (37°C) and pH (7) by testing a fixed amount of mycelium with increasing mycotoxin concentration.

**Figure 5:**
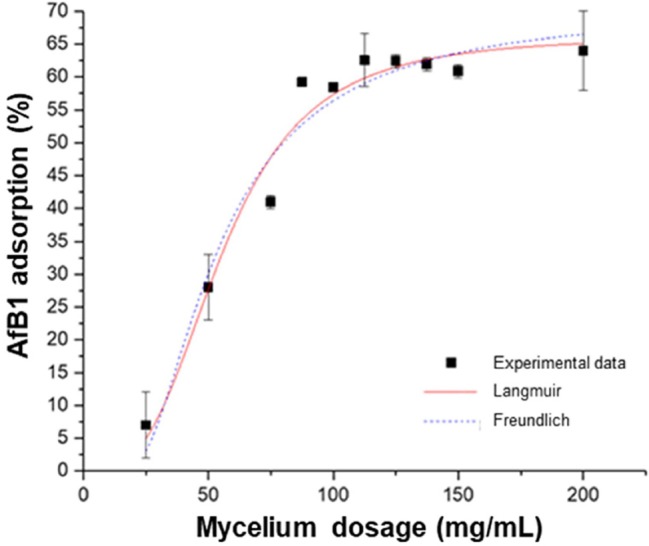
Effect of adsorbent dosage on AfB_1_ adsorption by mycelium. Equilibrium adsorption isotherms were obtained at constant temperature (37°C) and pH (7) by testing a fixed amount of mycotoxin with increasing adsorbent dosage.

Several adsorption isotherm models have been used to describe experimental adsorption data. The Langmuir and Freundlich models are the most frequently employed models. In this work, both models were used to describe the effect of mycotoxin concentration (Figure 4) and the effect of adsorbent dosage (Figure 5).

The linear regression analysis was applied to assess the goodness of the fits and to calculate the parameters involved in the adsorption mechanism (Table 4). The results obtained by comparing *R^2^* and SS_res_ showed that, for both the effect of adsorbent quantity and the effect of AfB_1_ concentration, the isotherm that fits the experimental data is the Langmuir isotherm. This suggests that the AfB_1_ adsorption mechanism is monolayer and that occurs at a finite (fixed) number of definite equivalent sites. The model describes a homogeneous adsorption in which each molecule has enthalpy and activation energy of the constant process and is graphically characterized by a plateau, such as a saturation point where each molecule occupies a site and there can be no further adsorption.

**Table 4:** Isotherm model parameters for the adsorption of AfB_1_ by *P. eryngii* mycelium.

Model	Parameter	Effect of AfB_1_ concentration	Effect of adsorbent dosage
Langmuir	K_L_ (±SE)	(4.3 ± 0.6)·10^−4^	(3 ± 7)·10^−6^
	q_m_ (±SE)	53 ± 19	66 ± 3
	*R*^2^	0.9976	0.9698
	SS_res_	16.47	100.70
Freundlich	K_F_ (±SE)	(50 ± 8)·10^−3^	70 ± 6
	1/n (±SE)	0.82	2.2 ± 0.5
	*R*^2^	0.9942	0.9580
	SS_res_	39.03	140.20

The Langmuir model can be used to predict whether the adsorption system is favorable or unfavorable by calculating the dimensionless constant K_R_ (Weber and Chakravorti, 1974). For favorable adsorption, the K_R_ value should fall in the range 0–1. The adsorption is considered unfavorable when K_R_ > 1, the isotherm is linear when K_R_ = 1, and the adsorption is irreversible when K_R_ = 0. In this study, the values of K_R_ for AfB_1_ adsorption on *P. eryngii* mycelium are comprised between 0 and 1, which suggests a favorable process for the system.

### Thermodynamic Parameters

The effect of temperature on the adsorption of AfB_1_ by *P. eryngii* mycelium was investigated. The uptake of AfB_1_ was found to increase when temperature increased: 66 ± 3% at 22°C, 67 ± 0% at 37°C, and 85 ± 3% at 50°C The increase of adsorption at increasing temperature indicates an endothermic nature of the adsorption process, as confirmed also by a positive ΔH^0^.

Molar free energy change of the adsorption process (ΔG^0^), standard enthalpy change (ΔH^0^), and standard entropy change (ΔS^0^) are shown in Table 5.

**Table 5:** Thermodynamic parameters for the AfB_1_ adsorption.

Temperature (°C)	lnK_0_	ΔG^0^ (kJ/mol)	ΔH^0^ (kJ/mol)	ΔS^0^ (kJ/mol K)
22	0.65	−1.59	30.62	0.11
37	0.71	−1,83		
50	1.77	−4.76		

The negative ΔG^0^ values are indicative of a spontaneous adsorption process. The ΔG^0^ values decreased as the temperature was raised, which is an indication of a physical adsorption nature of the process. Generally the free energy variation for the physical adsorption is between −20 and 0 kJ/mol, while in chemisorption, the range is −80 to 400 kJ/mol (Kavak, 2009). Besides the physical nature of the process, the experimental data show that the adsorption process needs to be activated by a moderately high temperature. This implies that the process is reversible and that the material can be regenerated by an appropriate treatment.

### Desorption Experiments

To verify the stability of the system over time, the percentage of desorption of the mycotoxin adsorbed on *P. eryngii* mycelium was assessed at room temperature and at the pH values of 3 and 7.4.

Desorption studies showed a very low desorption after 48 h at 25°C, at all the pH values tested. The percentage of desorption was 10 ± 4% at pH 3 and 7 ± 4% at pH 7.4. These results indicate a good stability of the system.

However, treatment with methanol resulted in a complete desorption of AfB_1_ from *P. eryngii* mycelium (recovery percentage 108 ± 6%). This result supports a possible re-utilization of the adsorbent after use, by regeneration of the adsorbing properties with an appropriate chemical treatment.

## Discussion

AfB_1_ is one of the most important mycotoxins. It is produced by different species of *Aspergillus*, mainly *A. flavus* and *A. parasiticus*, in a number of agricultural products, including cereals, wine, spices, flavor products, peanuts, and soy. In this research work, we studied a method for the removal of AfB_1_ from a solution by absorption, a promising detoxification technology that is growing in industrial interest and economic prospect. In particular, we investigated the AfB_1_-adsorbing capability of the fungal mycelium of *P. eryngii*, an edible mushroom. The mycelium was produced and then processed, making it enzymatically inert by sterilization at high temperatures and subsequent lyophilization. The material thus obtained was morphologically characterized by SEM and subjected to various batch tests to assess its performance as biosorbent.

The adsorbents for mycotoxins are high-molecular weight compounds that are able to bind mycotoxins in contaminated feeds without releasing them into the gastro-intestinal tract of the animal. In this way, the toxin-adsorbent complex passes through the animals’ intestine and is eliminated with the feces. This prevents or minimizes the exposure of the animal to mycotoxins (Kabak et al., 2006). The temperature and pH conditions during animal digestion vary according to the class they belong to. In particular for ruminants, which are polygastric animals (cattle, sheep), the bolus temperature is 38–40°C and the pH is 6.2–6.5. In the case of monogastrics, such as pigs, poultry, dogs, and cats, the pH varies during digestion from 4 to 6 and the temperature is between 38 and 40°C. For horses, the pH during digestion is 7.4–7.6 and the temperature is 37.5 –38.5°C. The biosorbent capability of *P. eryngii* mycelium was studied at the temperature of 37°C and at the pH values of 5 and 7, which are compatible with the temperature and pH of the gastro-intestinal apparatus of most farm animals (Cunningham and Klein, 2007). In contrast with the approach used in most of the studies on adsorbents, which is based on variation of one factor at a time, we applied a factorial design model (DOE) to evaluate the influence of the different operative factors on the biosorption process (Manal, 2007). Concentration of mycotoxin present in the solution and the quantity of adsorbent material were identified as determinants of the process. The pH of the solution was irrelevant in a range from 5 to 7 (range compatible with the pH of application). Zavala-Franco et al. (2018) studied the adsorbing capability of different biosorbents by an *in vitro* poultry digestive model. In that study, the same variables as in our work, that is mass of adsorbent and AfB_1_ concentration, were assumed to be the main variables affecting the system. Also, Phillips et al. (1988) and Diaz et al. (2002) reported that pH had no influence on AfB1-binding by inorganic adsorbents. The DOE method, that we adopted herein, is intended to describe the variation of outcomes under conditions that are hypothesized to reflect the variation. This mathematical approach was developed to extrapolate the information needed through the least number of independent experiments. The fact that the results of our study are consistent with those obtained by more traditional approaches corroborates the validity of the DOE approach.

The system works in the same way over a range of time that goes from 30 to 120 min. This allowed to obtain a system that reaches equilibrium in a very short time (30 min) and that was found to remain stable for 48 h at room temperature, at pH 3 and pH 7.4, giving a desorption of 10 ± 4% and 7 ± 4% respectively. In optimal conditions, the mycelium of *P. eryngii* reaches 85 ± 13% of Afb_1_ removal efficiency, values slightly lower than those achieved by other adsorbent materials, such as aluminosilicates (Phillips et al., 1988) and bentonites (Diaz et al., 2002), both of which can remove up to 95% of Afb_1_. However, the latter have the disadvantage of showing high inclusion rates for vitamins and minerals, while mycelium of *P. eryngii* can be used as an alternative adsorbent material that is effective without causing nutritional losses. In a recently reported study, the adsorbing capability of different biosorbents, i.e., banana peel, *Pyracantha* leaves, and *Aloe* powder, were compared to that of zeolite in a laboratory model that simulated the conditions of the poultry gastro-intestinal tract (Zavala-Franco et al., 2018). The adsorption values assessed were 70, 69, 46, and 28% for zeolite, *Aloe* powder, *Pyracantha* leaves, and banana peel, respectively. Although determined in a different experimental system and therefore hardly comparable, these values appear significantly lower than adsorption achieved with *Pleurotus* mycelium.

The value of ΔH^0^ of mycelium sorption is positive, indicating that the reaction is endothermic. The magnitude of ΔH^0^ gives an indication of the type of adsorption, which can be either physical or chemical (Della Gatta, 1985). In the first case, the energy requirement is small (<40 kJ/mol) allowing the equilibrium to be attained rapidly and the process to be easily reversible (Ringot et al., 2005). On the contrary, chemical adsorption involves higher enthalpy changes (>40 kJ/mol). In this study, the value of enthalpy is less than 40 kJ/mol, indicating a physical adsorption phenomenon. The positive and small value of ΔS^0^ reflects the little increasing randomness at the solid/liquid interface during the adsorption of AfB_1_ on *P. eryngii* mycelium. The reaction was reversible and optimization of the process resulted in 85 ± 13% of AfB_1_ removal.

The effectiveness of adsorption processes depends on the chemical structures of the adsorbent and the mycotoxin involved. The most important feature for adsorption is the physical structure of the adsorbent, that is, its total charge, the charge distribution, the pore size, and the accessible surface area. The properties of the adsorbed mycotoxins, such as polarity, solubility, shape, and charge distribution, also play a significant role (Huwig et al., 2001). To the best of our knowledge, there is no previously published study on the mycotoxin-binding capability of fungal mycelium, though the adsorbing capability of fungal biomass has been shown for several organic and mineral (heavy metals) pollutants (Ahmaruzzaman, 2008; Wang and Chen, 2009). To date, the biosorption mechanism of organic compounds and metal ions by fungal biomass has been studied largely in relation to chitin and its deacetylated derivative, chitosan. The carboxylate and/or phosphate ligands along with the hydroxy and amide functional groups on the fungal cell wall components, which form relatively weak bonds with adsorbed molecules, have been proposed to be involved. Our SEM observations showed that the cell walls of *P. eryngii* mycelium are highly porous, with a pore size of 5–15 μm, which significantly increases the exposure of the cell wall active surfaces and of the sites of binding, thus making the process more efficient.

## Conclusions

Our results show that non-viable mycelium of the fungus *P. eryngii* is able to efficiently adsorb AfB_1_ in conditions (temperature and pH) compatible with the physiology of animals’ digestion. A study was conducted to identify the major factors involved in the process. The concentration of mycotoxin in the solution and the quantity of adsorbent material were identified as determinants of the process. The pH of the solution was irrelevant in a range from 5 to 7 (range compatible with the pH of possible application). In addition, the system worked with no significant variation in the time lapse 30–120 min. of exposure. This allowed to obtain a system that reached equilibrium in a short time (30 min) and that remained stable in both acidic and slightly alkaline conditions that are compatible with pH values of the gastro-intestinal trait of farm animals. The thermodynamic study of the process showed that it is a spontaneous process with ΔG^0^ = −2.73 kJ/mol (average of ΔG^0^ at three temperatures 22, 37 and 50°C), endothermic (ΔH^0^ = 30.62 kJ/mol and ΔS^0^ = 0.11 kJ/mol·K) and that it is a physical adsorption, regulated by weak and reversible interactions, whereby the material can be regenerated with an appropriate treatment such as quantitative extraction with methanol. Optimization of biosorption resulted in 85 ± 13% of removal efficiency by *P. eryngii* mycelium.

The mycelium of *P. eryngii* is a biological and edible material and this characterizes this adsorbent as completely different from the materials currently used in the industry. The ongoing proof of concept and validation studies *in vitro* rumen models and *in vivo* might open the path for practical use of new, efficient though low-cost fungal mycelium-based feed additives for mycotoxin-biosorbtion and mitigation of mycotoxin risk.

## Data Availability

The datasets generated for this study are available on request to the corresponding author.

## Author Contributions

CA and MH conceived the research. CA, MH, and AL wrote the manuscript. MB carried out the microbiological work. EC carried out the DOE study. PA performed the scanning electron microscope (SEM) study. All authors designed the experiments, analyzed the data, contributed to manuscript revision, read and approved the submitted version.

### Conflict of Interest Statement

The authors declare that the research was conducted in the absence of any commercial or financial relationships that could be construed as a potential conflict of interest.

## Abbreviations

ΔG^0^: Gibbs free energy
ΔH^0^: Standard enthalpy
ΔS^0^: Standard entropy
ABTS: 2,2′-azino-bis-3-ethylbenzothiazoline-6-sulfonate
Ads%: Percentage of adsorption
AfB_1_: Aflatoxin B_1_
FLD: Fluorescence detector
LOQ: Quantification limit of the method
MEA: Malt extract agar
MEB: Malt extract broth
PBS: Phosphate buffer saline
SE: Secondary electrons
SEM: Scanning electron microscope.
